# Highly Sensitive Refractive Index Sensor Based on Polymer Bragg Grating: A Case Study on Extracellular Vesicles Detection

**DOI:** 10.3390/bios12060415

**Published:** 2022-06-15

**Authors:** Nabarun Saha, Giuseppe Brunetti, Arun Kumar, Mario Nicola Armenise, Caterina Ciminelli

**Affiliations:** 1 Optoelectronics Laboratory, Politecnico di Bari, Via E. Orabona 6, 70125 Bari, Italy; nabarun.saha@poliba.it (N.S.); giuseppe.brunetti@poliba.it (G.B.); marionicola.armenise@poliba.it (M.N.A.); 2 B-272, Prodhyogiki Apartments, Sec-3, New Delhi 110078, India; akumar@physics.iitd.ac.in

**Keywords:** photonic-crystals-based optical sensing, refractive index sensing, optical biosensing, waveguide-based sensing

## Abstract

The measurement of small changes in the refractive index (RI) leads to a comprehensive analysis of different biochemical substances, paving the way to non-invasive and cost-effective medical diagnosis. In recent times, the liquid biopsy for cancer detection via extracellular vesicles (EV) in the bodily fluid is becoming very popular thanks to less invasiveness and stability. In this context, here we propose a highly sensitive RI sensor based on a compact high-index-coated polymer waveguide Bragg grating with a metal under cladding. Owing to the combined effect of a metal under cladding and a high-index coating, a significant enhancement in the RI sensitivity as well as the dynamic range has been observed. The proposed sensor has been analyzed by combining finite element method (FEM) and coupled-mode theory (CMT) approaches, demonstrating a sensitivity of 408–861 nm/RIU over a broad dynamic range of 1.32–1.44, and a strong evanescent field within a 150 nm proximity to the waveguide surface compliant with EV size. The aforementioned performance makes the proposed device suitable for performing real-time and on-chip diagnoses of cancer in the early stage.

## 1. Introduction

Refractive index sensors based on lab-on-chip photonic structures have gained a lot of attention in recent times and are becoming a paramount tool as they are compact, immune to electromagnetic interference, and amenable to integration with other photonic components in a single chip as well as with microfluidic channels [1,2,3]. Therefore, highly sensitive RI sensors based on photonic structures lead us to the cost effective, rapid, and precise detection of substances, making them suitable for environmental monitoring, food quality control, and especially medical diagnosis such as detecting bacterial infections, cancer, disorders, various physical and biological parameters to examine and treat health conditions, etc. [4,5,6,7]. In recent times liquid biopsies are becoming a popular medical diagnosis technique to detect and monitor cancerous tumors through the detection of different biomarkers such as circulating tumor cells (CTCs), circulating tumor nucleic acids ctNAs (ctDNA, microRNAs), as well as extracellular vesicles (EVs) [8]. Considering that these biomarkers are present in bodily fluids such as blood, saliva, and urine, a liquid biopsy is a less invasive process than a tissue biopsy, making it less painful and also reducing the chances of infections [9,10]. Among the aforementioned biomarkers, EVs are stable membrane particles secreted by cells, containing the molecular imprint of proteins, lipids, and nucleic acids [11]. In recent times they have been drawing particular attention due to their stability and their presence in bodily fluids in the early stages of cancer. In addition, the expressions of EVs in bodily fluids are also found to be promising for monitoring different diseases including neurodegenerative [8], cardiovascular [12], and various infectious ones [13]. In view of this, recently few techniques have been reported in the literature based on electrochemical and photonic structures, which shows the potential of EVs in detecting and monitoring cancer in the early stage [14,15,16]. The proposed structures also show that the detection of EVs depends on the suitable bio-recognition elements as well as RI sensitivity as the presence of EVs cause a considerable change in sample RI. Therefore, it is imperative to design a photonic sensor structure, which shows a high RI sensitivity with a compact footprint and a low loss for real-time, label free, and efficient detection of cancer through EV biomarkers.

The RI sensitivity of photonic structures depends on the interacting evanescent field of the guided modes with the sensing medium covering the waveguide surfaces. The high interacting field leads to a high sensitivity. Over the years, various photonic structures have been proposed such as surface Plasmon polaritons (SPPs) [17,18], 2D materials [19,20], long period gratings (LPGs) [21,22], multimode interferences [23], ring resonators [24,25], Bragg gratings [26,27,28,29,30,31], etc. The performance of the structures based on LPGs, SPPs, multimode interferometers, and ring resonators is often limited by broad bandwidth, a bulky setup, or a free spectral range. On the other hand, a Bragg grating is free from the above-mentioned issues and has a simple straightforward robust structure with a narrow band response. In Bragg gratings, the forward propagating fundamental mode couples to the counter-propagating fundamental mode that is well confined within the waveguide core. As a result, the sensitivity associated with the Bragg gratings is usually inferior to the reported sensitivity of the structures based on the other architectures. Thus, the enhancement of the interacting field with the sensing medium in Bragg gratings can lead to very efficient on-chip photonic sensors. In order to address this issue, a few techniques have been reported in last two decades by different research groups. For example, Dai etal. [26] have reported an open top ridge to expose the fundamental mode directly into the ambient medium to enhance the sensitivity. The RI sensitivity is found to be between 25–33 nm/RIU with an aqueous medium (RI = 1.33) as surroundings. A further enhancement in sensitivity has been achieved by Tripathi et al. [27], utilizing the discontinuity of a modal field at the surface of a high-index-contrast silicon waveguide that yields a sensitivity of 239 nm/RIU. However, an extra photosensitive cladding layer has been considered on top of the silicon waveguide to form the grating that hinders the participating mode from directly interacting with the ambient medium. In order to further expose the core mode to the sensing medium, Sub Wavelength Grating (SWG) geometry has been utilized with laterally loaded blocks as the Bragg grating [28,29]. In SWGs, in addition to the evanescent field, a part of the guiding power in the core also interacts with the sensing medium. Following this structure, a high sensitivity of 507 nm/RIU has been reported recently [29]; however, the requirement of a double periodicity (SWG and Bragg grating) and the periodical loading of blocks to form the gratings also brings complexity in device geometry. Earlier we have reported a Bragg grating-based sensor with metal under cladding in simple ridge waveguide geometry [30]. It has been observed that the metal under cladding can significantly enhance the evanescent field in the sensing medium for the non-plasmonic mode and yields a sensitivity of 237–578 nm/RIU for an RI range of 1.3–1.4, suitable for bio-sensing. In addition, unlike the previous structures the proposed sensor does not require any waveguide birefringence control or any analyzer/polarizer due to the large differential modal losses of the TE and TM modes. However, the sensor structure suffers from few issues such as the device cost due to the gold metallic layer and the poor adhesion between the germanosilicate core and the metal layer. In one recent study, authors have reported that a high-index coating on a polymer waveguide can also significantly improve the RI sensitivity [31]. The reported sensitivity is found to be higher (169–527 nm/RIU) in an RI range of 1.4 to 1.59, slightly away from the typical biosensing RI range. In this paper, a combined phenomenon of metal under cladding and a high-index coating has been reported in polymer ridge waveguide geometry with the aim of the further advancement of RI sensitivity in the biosensing range. The sensitivity is found to be quite high, varying between 408 to 861 nm/RIU in a broad RI range of 1.32 to 1.44. The proposed sensor is also found to be cost effective and easy to realize practically. Further, it has been noticed that due to the high-index coating, the enhancement in the evanescent field is quite high within 150 nm of waveguide surface, making it an excellent choice for EV detection as the typical size of EVs fall within this.

## 2. Sensor Configuration and Modeling

**Figure 1:**
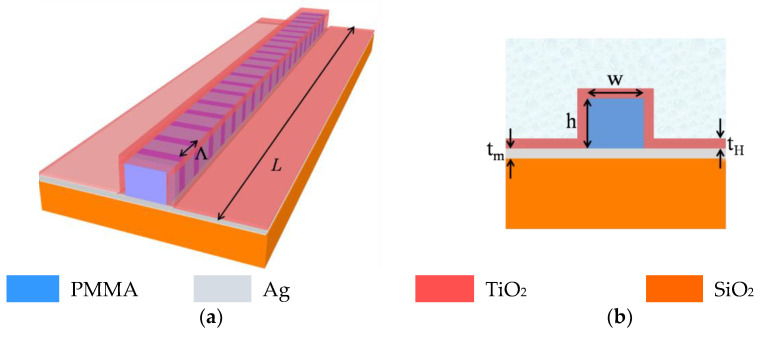
(**a**) 3D schematic and (**b**) cross sectional view of the proposed sensor.

The schematic of the proposed sensor is shown in Figure 1 in which (a) represents the 3D view whereas (b) illustrates the cross-sectional view. It consists of a polymer core made of Polymethyl methacrylate (PMMA) with a thin metallic layer of silver (Ag) underneath the core having a thickness *t_m_*. It is worth noting that, unlike the germanosilicate core in Ref. [30], here we have considered PMMA as the waveguide core since it shows a good adhesion with the metal layer. Further, PMMA can directly act as the resist as well as waveguide core, which makes it easy to form the ridge waveguide using deep UV lithography or e-beam lithography, which has been reported with well-known dielectric-loaded surface Plasmon polariton (DLSPP) waveguide geometry [32]. The entire structure is considered to be coated with a nano-layer of titanium dioxide (TiO_2_) having a thickness *t_H_*, which can be deposited following the sputtering technique [29,33]. Since the entire structure is coated with the high-index layer, the metal layer is not exposed to the ambient medium. As a result, silver has been considered as the metal layer instead of gold, which can significantly reduce the device cost, making it suitable for mass production.

The Bragg grating is considered to be written in the PMMA core as a periodic modulation of the refractive index having periodicity *Λ* and length *L*. Taking advantage of PMMA’s photosensitivity to UV radiation, the Bragg grating can be formed using periodic exposure to a UV laser [34,35]. It is worth noting that the reflection by the Ag layer at two sides of the waveguide core can be a source of concern while writing the grating. Therefore, a properly designed mask can be used on top of the waveguide core to expose only the PMMA core of the waveguide to the UV laser [34]. The analyte itself acts as upper cladding of the sensor geometry.

**Figure 2:**
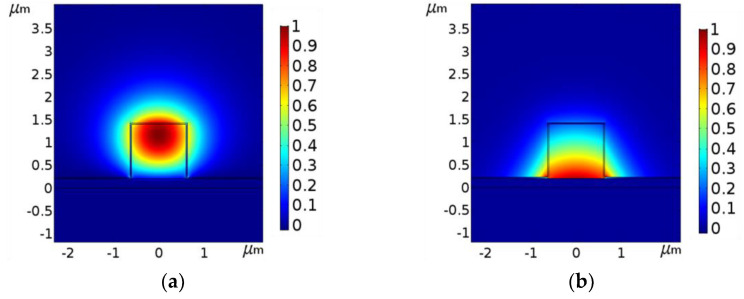
Major field component (**a**) *E_x_* of TE_00_ mode and (**b**) *E_y_* of TM_00_ mode. The waveguide parameters are *w* = 1.2 μm, *h* = 1.2 μm, *t_m_* = 200 nm, and *t_H_* = 30 nm.

The modal field distribution of the major field component *E_x_* (*E_y_*) of the TE_00_ (TM_00_) mode is shown in Figure 2, the modal effective index of which is 1.3833 + 8.13339 × 10^−5^*i* (1.4712 + 6.8074 × 10^−4^*i*). The waveguide parameters are considered to be *w* = 1.2 µm, *h* = 1.2 µm, *t_m_* = 200 nm, *t_H_* = 30 nm, a detailed discussion about selecting such parameters is given in the next section. The mode analysis of the proposed sensor has been carried out using the fully vectorial finite element method (FEM) using COMSOL multiphysics. Figure 2 shows that the evanescent field associated with the TE_00_ mode is quite high in the ambient medium whereas the TM_00_ mode is mostly confined in the core adjacent to the metal layer. The fractional modal power (FMP) in the ambient medium is found to be 37.6% for the TE_00_ mode whereas it is 21.8% for the TM_00_ mode.

Further, the imaginary part of the TE_00_ mode effective index is one order lower than that of the TM_00_ mode. As a result, the propagation length *L_p_* (distance at which power becomes 1/e to that of the input) of the TE_00_ mode (1.51 mm) is much higher as compared to the TM mode (0.18 mm). In view of these two factors, the analysis has been carried out considering the TE_00_ mode, which is the non-plasmonic mode of the structure.

The forward propagating TE_00_ mode couples to the backward propagating TE_00_ mode via the grating and gives rise to the resonance peak in the reflected spectrum at the phase matching condition $\lambda_{R}{=2n}_{0,0}\Lambda$, where $\lambda_{R}$ and $n_{0,0}$ is the resonance wavelength and modal effective index of the TE_00_ mode, respectively. The coupled mode theory (CMT) yields the reflected spectrum governed by the equation [29,30].

(1) $$R={\left|\frac{\kappa \tan h(\gamma L)}{\gamma +(\alpha +i\delta )\tan h(\gamma L)}\right|}^{2}$$

where $\delta =\frac{\pi }{\lambda }{(2n}_{0,0}\;-\;\frac{\lambda }{\Lambda })$ is the phase mismatch factor, α is the loss coefficient, and $\gamma =\sqrt{{\left(\alpha +i\delta \right)}^{2}{+\kappa }^{2}}$ in which κ is the coupling coefficient given by [30],

(2) $$\kappa =\frac{n_{\mathrm{co}}{\omega \varepsilon }_{o}}{2}\iint \Delta nE.E\mathrm{dA}$$

In the above equation, *n_co_* represents the RI of the waveguide core, ∆*n* is the grating strength, i.e., the amplitude of RI modulation and **E** is the power normalized electric field. At the phase matching wavelength, the reflectivity reaches its peak value, which can be obtained from Equation (1) by putting the condition δ=0 which comes out to be,

(3) $$R_{\max}={\left|\frac{\kappa \tan h(\sigma L)}{\sigma +\alpha \tan h(\sigma L)}\right|}^{2}$$

where, $\sigma =\sqrt{\alpha^{2}{+\kappa }^{2}}$. The sensitivity of the proposed structure (S) has been calculated following the shift of resonance wavelength with respect to the change in the ambient medium’s RI that can be expressed as [29],

(4) $$S=\frac{{d\lambda }_{R}}{{dn}_{a}}=\frac{\lambda_{R}}{n_{g}}\left(\frac{\partial n_{0,0}}{\partial n_{a}}\right)$$

where, *n_a_* represents the ambient RI and $n_{g}{=n}_{0,0}\;-\;\lambda \frac{\partial n_{0,0}}{\partial \lambda }\;$denotes the group index of the fundamental mode. In order to account for the wavelength dependency on the RIs of the waveguide materials, the corresponding Sellmeier relations has been used for the silica substrate [30], the PMMA core [36], and the TiO_2_ coating [37], whereas for the silver layer Johnson and Christy’s data has been considered with a cubic and linear fit for real and imaginary part of refractive index, respectively, as reported in [38,39].

## 3. Results and Discussions

### 3.1. Design and Performance Estimation

**Figure 3:**
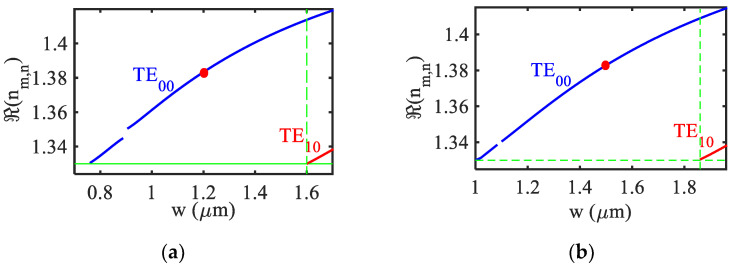
Variation of the real part of effective index of TE_00_ mode and first higher-order mode TE_10_ as a function of square waveguide’s width (w) for (**a**) HI-MCRW and (**b**) MCRW. The red dots represent the middle point of the single mode region.

In order to understand the effect of the high-index coating on sensitivity, the proposed high index coated metal clad ridge waveguide (HI-MCRW) structure has been compared with the metal clad ridge waveguide (MCRW) without the high-index coating. In both of the cases, a square cross-section (*w* = *h*) has been considered with PMMA being the core material. Since for the Bragg grating-based structure the single mode (SM) operation is preferred, first, the SM region for both the geometries has been identified for the TE mode. In Figure 3a,b the real part of the modal effective index of the TE_00_ mode and the first higher order mode TE_10_ has been plotted as a function of square cross section’s width w for HI-MCRW and MCRW respectively.

The figure shows that the SM region is 0.76 µm to 1.6 µm for the HI-MCRW structure whereas it is 1 µm to 1.86 µm for the MCRW. The thickness of the metal layer is taken to be 200 nm to reduce the power leakage in the substrate [21]. It is worth noting that with the increase in the high-index layer thickness, although the power in the ambient will increase, the power in the core will also decrease, leading to the reduction in coupling strength and thus a longer grating length. Therefore, the thickness of the high-index TiO_2_layer is considered to be 30 nm. The grating length is taken to be 0.8 mm such that it is lower (higher) than the propagation length 1.51 mm (0.18 mm) of the TE(TM) mode. This ensures that the power associated with the TM mode is negligible at the output, which dismisses the requirement of a polarizer to separate the TE and TM modes.

In order to compare the device performance with and without the high-index layer, the waveguide’s core dimension for HI-MCRW and MCRW are considered to be 1.2 × 1.2 µm and 1.5 × 1.5 µm such that both of them are at the middle of their respective SM regions as marked with red dots in Figure 3. It is worth noting that the discontinuity present in each graph in Figure 3 can be attributed to the mode hybridization (MH) phenomenon that has been widely studied in silicon and lithium niobate waveguide geometries [40,41]. In the proposed sensor it plays an important role in peak reflectivity, thus a careful device design has been performed following the below discussion. In MH, the E_x_ and E_y_ field component of the quasi-TE and TM mode becomes comparable and therefore the mode polarization cannot be distinguished. Here the MH takes place between the TE_00_ and TM_10_ modes, the strength of which can be measured in terms of the hybridization factor defined as [40],

(5) $$\eta =\frac{\iint E_{x}^{2}\mathrm{dA}}{\iint E_{x}^{2}\mathrm{dA}+\iint E_{y}^{2}\mathrm{dA}}$$

**Figure 4:**
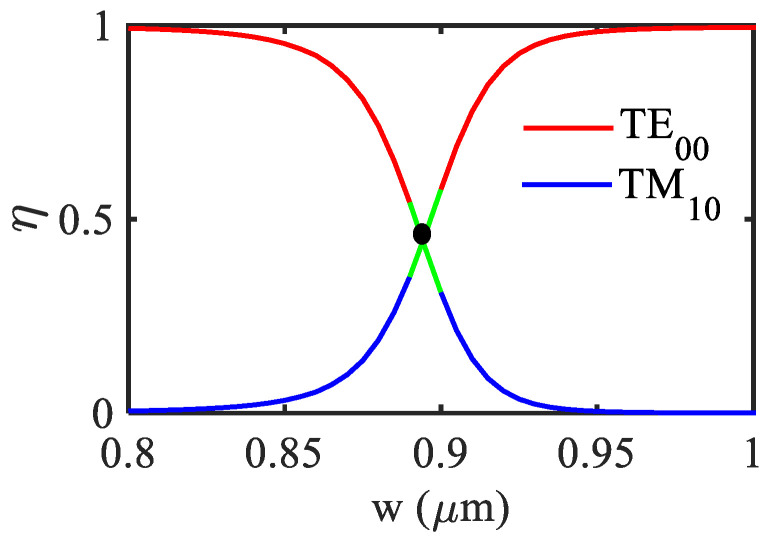
Variation of mode hybridization factor with waveguide’s width for HI-MCRW.

In Figure 4 the hybridization factor (η) has been plotted as a function of the waveguide’s width for the HI-MCRW structure. In the figure, the blue and red curve, respectively, represents the TE_00_ and TM_10_ modes. At *w* = 0.89 µm, hybridization factor becomes 0.5, highlighting that the contribution coming from the E_x_ and E_y_ component for both the modes are equal. Since Figure 3 is plotted for the TE mode of the structure and at *w* = 0.89 µm the polarization of the mode cannot be distinguished, a discontinuity in the graph originated. The range of the discontinuity has been decided by the behavior of the propagation length *L_p_* with respect to the grating length as discussed below.

**Figure 5:**
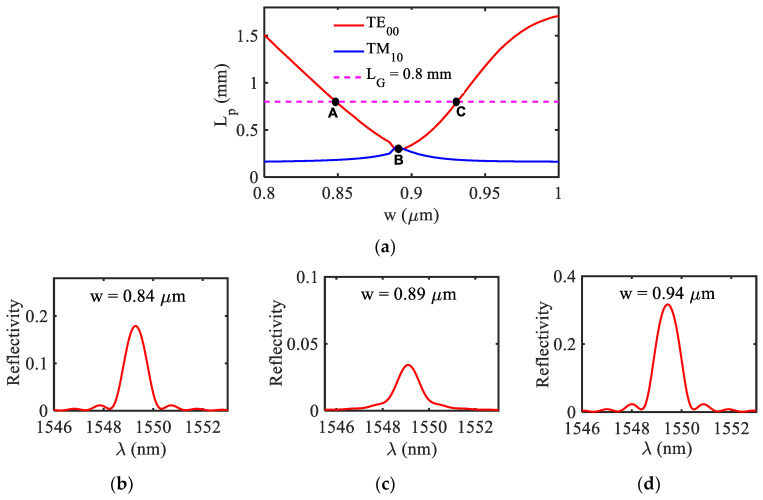
(**a**) Propagation length of TE_00_ and TM_10_ mode as a function of width of HI-MCRW. Reflected spectrum of HI-MCRW corresponding to TE_00_ mode for three different widths (**b**) *w* = 0.84 µm, (**c**) *w* = 0.89 µm, (**d**) *w* = 0.94 µm as represented by three different points A, B, and C in (**a**).

It has been noticed that around the hybridized point, the modal loss associated with the TE_00_ mode is quite high, which affects the peak reflectivity of the proposed sensor. In order to show this, in Figure 5a we have plotted the propagation length *L_p_* as a function of the HI-MCRW’s width (*w*) for both the modes and in Figure 5b–d, respectively, the reflected spectrum for three different widths corresponding to points A, B, and C as highlighted in Figure 5a. The dotted horizontal line in Figure 5a represents the grating length 0.8 mm.

At point B (hybridized point), since the propagation length of the mode is much lower than the grating length, the peak reflectivity is extremely small (< 0.05), making it difficult to detect. At points A and C, the peak reflectivity is significantly higher as the propagation length of the mode equal to the grating length. A similar kind of behavior also observed with the ambient RIs. In view of the above, from a practical point of view, in our analysis we have neglected the region around the hybridized point where the propagation length is lower than the grating length. Therefore, the range of discontinuity in Figure 3 represents the region in which the propagation length of the TE_00_ mode is lower than the grating length.

**Figure 6:**
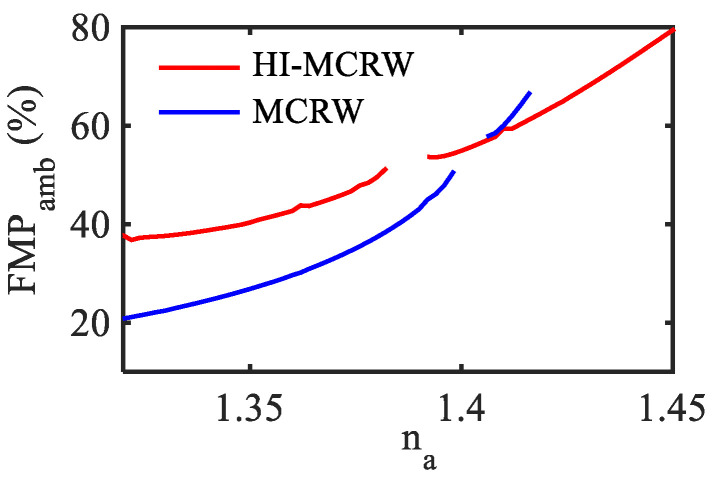
FMP at the ambient medium for the TE_00_ mode as a function of its RI for HI-MCRW and MCRW.

Considering the aforementioned features, in Figure 6 the variation of FMP in the ambient medium as a function of ambient RI (*n_a_*) has been presented for both the structures at wavelength 1.55 µm. The FMP in the ambient medium is defined as,

(6) $$FMP_{amb}=\frac{\iint_{Ar}^{}E\times HdA}{\int_{-\infty }^{\infty }\int_{-\infty }^{\infty }E\times HdA}\;$$

where, *Ar* represents the area covering the ambient medium whereas ***E*** and ***H*** are the electric and magnetic field components of the TE_00_ mode. In both cases, the graph has been plotted up to the cut-off point of the TE_00_ mode with respect to the ambient (cover) RI that is found to be 1.45 and 1.416 for HI-MCRW and MCRW, respectively. The discontinuity in the graphs occurred due to the MH as mentioned earlier and the range of discontinuity is highlighting the range of ambient RI for which the propagation length is lower than the grating length around the MH. The FMP is found to be mostly in the higher side for the HI-MCRW structure as the high-index coating pulls the guided mode towards the ambient medium. Near the mode’s cut-off, the FMP is found to be 79.4% for the HI-MCRW whereas it is 66.5% for the MCRW. The FMP at the ambient RI = 1.32 is 37.7% and 20.7% for HI-MCRW and MCRW, respectively, showing a better enhancement at a lower RI as well.

**Figure 7:**
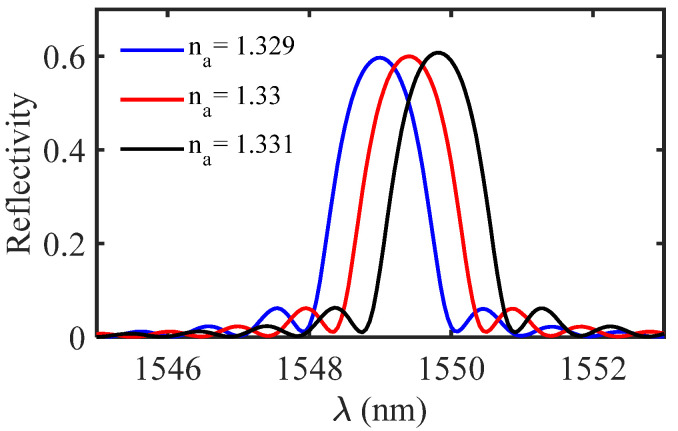
Reflected spectrum of the proposed HI-MCRW structure for three different ambient RIs.

The reflected spectrum of the proposed HI-MCRW has been shown in Figure 7 for three different ambient RIs. The grating period is considered to be 560 nm, such that the resonance wavelength is around 1.55 µm, whereas the grating strength is taken to be 8 × 10^−4^. The spectrum shows a red shift in the resonance wavelength with the increase in ambient RI.

**Figure 8:**
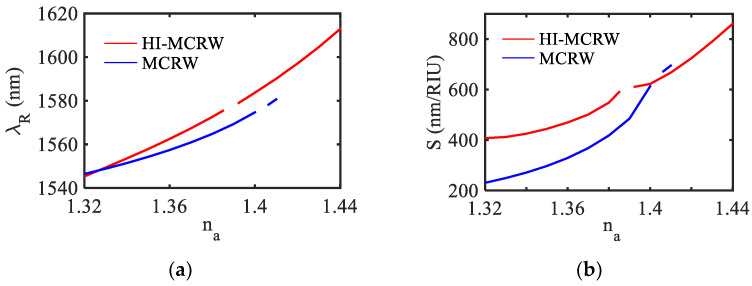
(**a**) Variation of resonance wavelength and (**b**) corresponding RI sensitivity with ambient RI for the TE_00_ mode.

The corresponding resonance wavelength and the sensitivity have been shown in Figure 8a,b, respectively, as a function of ambient RI (*n_a_*) up to the cut-off point for both structures. Again, it is worth noting that the discontinuity in the graphs is due to the MH phenomenon. The overall shift in the resonance wavelength is found to be 67.6 nm for the proposed HI-MCRW structure, which is nearly twice that of 34.2 nm corresponding to the MCRW.

In view of the fact that the shift in the resonance wavelength is non-linear, the sensitivity has been calculated at each ambient RI following Equation (4), which is presented in Figure 8b. The sensitivity is found to vary between 407.7 to 860.6 nm/RIU for an RI range of 1.32 to 1.44 for the proposed HI-MCRW structure, whereas it varies from 230.7 nm/RIU to 694.8 nm/RIU for the RI range of 1.32 to 1.41 in the absence of the high-index coating. It is important to note that the high-index coating also increases the dynamic range in addition to the enhancement in sensitivity. The increment in the dynamic range can be attributed to the increase in the modal effective index owing to the presence of the high-index coating, allowing the proposed structure to reach its cut off at a higher ambient RI. Apart from the sensitivity and dynamic range, another important parameter to characterize the device performance is the figure of merit (FOM), defined as [30],

(7) $$\mathrm{FOM}=\frac{\mathrm{Sensitivity}\;(\mathrm{nm}/\mathrm{RIU})}{\mathrm{FWHM}\;(\mathrm{nm})}$$

where, FWHM represents the full width at half maxima of the reflection peak. The FOM is found to be 308.2 RIU^−1^ and 143.6 RIU^−1^ for the HI-MCRW and MCRW, respectively, showing a more than two times enhancement due to the high-index coating. Considering the spectrometer resolution to be 20 pm, the detection limit is found to be 4.8 × 10^−5^ RIU and 8×10^−5^ RIU for the HI-MCRW and MCRW, respectively, around the ambient RI = 1.33.

In the following table (Table 1), we have compared the sensitivity and dynamic range along with the corresponding operating principles of different reported structures with Bragg grating geometry, highlighting the good performance of the proposed grating geometry. In addition, the highly lossy nature of the TM modes ensures a polarizer/analyzer and birefringence control-free operation of the proposed structure, which gives it an edge over the other structures as well.

### 3.2. EV Detection: A Case of Study

As mentioned earlier, owing to their stability and presence in bodily fluids, the EVs are a promising biomarker for the detection of cancer [42] in their early stages as well as various infectious diseases, cardiovascular diseases, etc. Despite these beneficial features, the detection of EVs with a high sensitivity and a high accuracy is still a challenge. In order to enhance the sensitivity, different techniques based on electrochemical [14], plasmonics [15,43], and photonics [16,44] have been proposed in recent times. Although electrochemical sensors show a good sensitivity and accuracy, their applications are often limited as it is difficult to realize sensor multiplexing [16]. The plasmonic-based sensors can overcome this, and it also shows a high sensitivity owing to the presence of a large evanescent field at the metal-sensing medium interface, but the intrinsic loss due to the metal layer often leads to a high signal to noise ratio and thus less accuracy. In this context, the proposed sensor can be a suitable one since the high RI sensitivity has been achieved with a non-plasmonic mode of the structure that shows a one order less propagation loss as compared to the plasmonic mode. Further, it has been observed that due to the high-index coating, the FMP within the 150 nm vicinity of the waveguide surface is found to be around 13% for the HI-MCRW, whereas it is 6.7% for the MCRW in the middle of their respective single mode regions. This enhancement in FMP due to the high-index nano-layer is particularly promising for the EV detection as the typical size of EVs is 100±20 nm [16].

**Figure 9:**
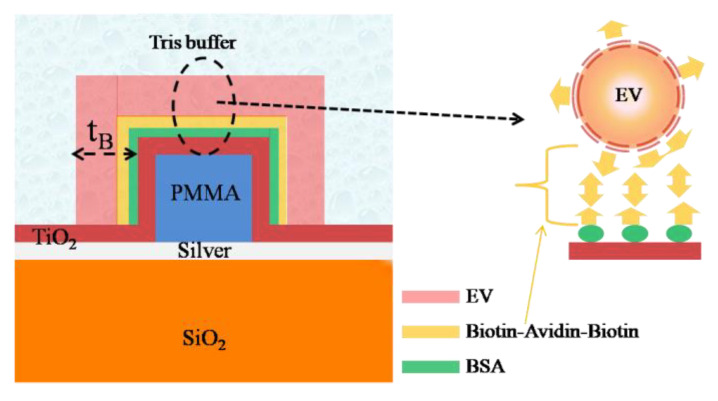
Cross sectional view of the proposed sensor functionalized for EV sensing. The left side image represents enlarged view of the model used for EV detection.

**Figure 10:**
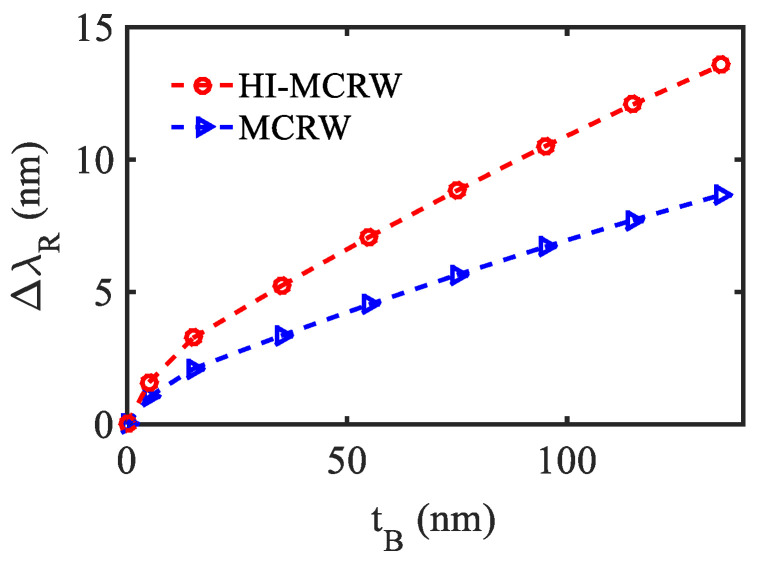
Shift in resonance wavelength for the HI-MCRW and MCRW corresponding to EV detection.

In view of this, in this section, the proposed sensor has been modeled to investigate its potential for the EV detection following the recently reported technique in Ref. [16]. The surface geometry of the proposed structure functionalized for EV detection is shown in Figure 9. First, the waveguide surface has been functionalized with a biotinylated layer consisting of bovine serum albumin (BSA) and biotin followed by another layer of avidin. The EVs are also considered to be biotinylated such that the strong affinity between the biotin and avidin can be utilized to detect EVs with a good selectivity as reported in [16]. In the numerical simulation, the thickness and the RI of the BSA layer has been considered to be 5 nm and 1.57, respectively [45,46,47]. Next, a single layer of biotin–avidin–biotin is considered to have a thickness of 10 nm and RI = 1.45 [48,49]. Eventually, a layer of EV having of RI = 1.398 [50] has been considered as the final layer. The entire structure is considered to be covered with Tris buffer medium, the RI of which has been obtained using the relevant Sellmeier relation [51]. The shift in the resonance wavelength (λ_R_) has been plotted in Figure 10 for both structures as a function of the bio-layers thickness (t_B_), considering the aforementioned waveguide parameters. The initial shift in λ_R_ is due to the BSA layer and biotin–avidin–biotin layer that are found to be 3.25 nm and 2.1 nm for the HI-MCRW and MCRW, respectively. Next, the shift in λ_R_ occurs due to the EVs that show a linearly increasing characteristic. The total shift in resonance wavelength for the HI-MCRW is found to be 13.6 nm, whereas it is 8.7 nm for the MCRW structure, highlighting an approximate5 nm higher shift due to the high-index coating. Considering that the enhancement in wavelength shift can be helpful in detecting EVs with lower concentrations, the proposed sensor holds a great promise for early-stage cancer detection.

### 3.3. Fabrication Tolerances and Readout Concept

For the practical realization of a device, it is important to know the fabrication tolerances of different structural parameters. Therefore, following the well-established fabrication techniques, the effect of any inaccuracy in waveguide’s parameters on two important quantities: the sensitivity and peak reflectivity have been investigated around an ambient RI of 1.33. The inaccuracy in a waveguide’s width is considered to be ±10 nm, which is usually ensured by the standard e-beam lithographic technique [52,53]. The corresponding variation in sensitivity and peak reflectivity is found to be within 5 nm/RIU and 0.016 from the respective value of 412 nm/RIU and 0.6 at the nominal width of 1.2 μm, reflecting a good tolerance with respect to the width. The sensitivity shows a stable performance for the same order of variation in the waveguide’s height whereas the variation in peak reflectivity is found to be well within 0.014. It is worth noting that, for the considered metal layer thickness of *t_m_* = 200 nm, the modal power in the core as well as in ambient is well saturated [21,30]. As a result, the same order (±10 nm) of variation in *t_m_* results in an ignorable deviation in sensitivity and peak reflectivity. However, the proposed structure shows a less forbearing performance with respect to any deviation from the nominal value of 30 nm of the high-index layer thickness *t_H_*. For example, a ±5% and a ±10% inaccuracy in *t_H_* (results in an error of ±1.5 nm and ±3 nm) causes a 12 nm/RIU and 30 nm/RIU deviation in sensitivity, respectively, whereas a 0.7 and 0.15 deviation in peak reflectivity, respectively. Although popular deposition techniques such as sputtering can provide an approximate ±1.5 nm accuracy [54,55], proper care should be taken while depositing the high-index TiO_2_ layer. For the readout, single mode optical fiber pigtails can be butt-coupled with the proposed structure [31] in order to integrate it with a broadband source (ASE) [56] and an optical spectral analyzer to detect and monitor the reflected spectrum [56]. As mentioned earlier, due to the highly differential propagation losses of the TE and TM mode, in the readout, the polarization control is not necessary for the proposed structure.

## 4. Conclusions

A cost-effective, compact, and highly sensitive RI sensor is proposed with a high-index coated Bragg grating-inscribed polymer ridge waveguide with a metal layer underneath the core. The combination of a high-index coating and a metal under cladding yields a significant amount of evanescent field to interact with the sensing medium associated with the non-plasmonic mode, resulting in a high RI sensitivity of 408–861 nm/RIU in an RI range of 1.32–1.44. In addition, the high-index coating is also found to be beneficial in enhancing the dynamic range of the sensing medium. The proposed structure is found to be an excellent match for EV detection thanks to the enhanced evanescent field in the 150 nm vicinity of the waveguide surface due to the high-index coating, which matches well with the typical size of EVs. Owing to this enhancement and provided that the non-plasmonic mode is involved in accomplishing a high sensitivity, the proposed structure should be able to detect a low concentration of EVs with a good accuracy, leading to a promising development towards early-stage cancer detection.

## Figures and Tables

**Table 1:** Comparison of the proposed sensor with previously reported Bragg grating-based sensors.

Ref.	S (nm/RIU)	Dynamic Range (RIU)	Operating Principle
[26]	7–25	1.3–1.402	Open top ridge waveguide
[27]	200–740	1.33–1.63	SOI with photosensitive upper cladding
[29]	507	1.3–1.34	Sub-wavelength gratings
[30]	237–578	1.3–1.4	Metal clad ridge waveguide
[31]	169–523	1.4–1.59	High-index coated ridge waveguide
Proposed structure	408–861	1.32–1.44	High-index coated polymer waveguide with metal under cladding
